# Assessing the Color and Surface Characteristics of Additively Fabricated Denture Base Resins Containing Nanoparticles

**DOI:** 10.1155/ijod/5177847

**Published:** 2025-07-25

**Authors:** Shaimaa M. Fouda, Mohammed M. Gad, Mai Salah El-Din, Soban Q. Khan, Ahmed Othman, Constantin von See

**Affiliations:** ^1^Department of Substitutive Dental Sciences, College of Dentistry, Imam Abdulrahman Bin Faisal University, Dammam, Eastern Province, Saudi Arabia; ^2^Department of Prosthodontics, Alexandria University Main Hospital, Alexandria, Egypt; ^3^Department of Dental Education, College of Dentistry, Imam Abdulrahman Bin Faisal University, Dammam, Eastern Province, Saudi Arabia; ^4^Research Center for Digital Technologies in Dentistry and CAD/CAM, Department of Dentistry, Faculty of Medicine and Dentistry, Danube Private University, Krems, An Der Donau, Lower Austria, Austria

**Keywords:** 3D printing, complete denture, denture base resin, hardness, optical properties, surface roughness

## Abstract

**Objective:** To investigate and compare the impact of different nanoparticles (NPs) on the color stability and surface properties of additively fabricated (AF) denture base resins after thermal cycling.

**Materials and Methods:** Two hundred specimens (*n* = 10) were AF from NextDent and ASIGA denture base resins. Specimens were divided into four groups according to the added NP's type and concentration: nanodiamond (ND; 0.25% and 0.5%), silicon dioxide NPs (NS; 0.25% and 0.5%), and a control group of pure resin of each material. The color change (∆*E*_Lab_), surface roughness, and hardness were tested after thermal cycling (5000 cycles). Data were analyzed using ANOVA and post hoc Tukey's tests (*α* = 0.05).

**Results:** The ∆*E*_Lab_ was affected by the NP type and material type, while the NPs concentration showed no effect. ND showed higher ∆*E*_Lab_ with NextDent in all groups (*p*  < 0.05) in comparison to NS. Color change of ASIGA with ND and NS was higher than that of NextDent, ∆*E*_Lab_ values of all tested groups were below 3.7 NBS. For NextDent, 0.5% NS showed significantly the highest hardness values (25.9 ± 4.6 VHN) followed by 0.5% ND (18.7 ± 2.4 VHN). For hardness, NextDent 0.5% NS showed significant increase when compared with ASIGA (*p*  < 0.001). For roughness, NextDent 0.5% NS showed significantly less Ra than other groups. In term of materials comparison per NP type, significant differences in Ra between ASIGA and NextDent was detected.

**Conclusions:** The effect of NP on the tested properties is concentration dependent and material dependent therefore, NP type, NP concentrations, and material type selection should be considered for AF nanocomposite denture base fabrication.

## 1. Introduction

The use of 3D printing technology in denture fabrication has increased due to multiple factors including the ease of fabrication with few clinical appointments, increased patient satisfaction and low cost [1]. However, additively fabricated (AF) dentures lack the mechanical strength of milled dentures [2, 3]. Some studies reported comparable biocompatibility and surface roughness of AF compared to milled resin [4]. Nevertheless, the mechanical properties of AF denture base resin were found inferior to heat polymerized polymethyl methacrylate (PMMA) [5].

Many factors could improve the mechanical performance of AF resin amongst is the addition of fillers or nanofillers to the resin [6, 7]. The addition of nanoparticles (NPs) to denture base resin improved the mechanical properties of heat, auto, and microwave cured PMMA [8]. NPs also improved the mechanical performance of AF denture base resin [9].

Silicon dioxide NPs (NS) was tested in previous studies and proved enhancement of the mechanical properties of conventional and AF denture base materials [10, 11]. Nanodiamonds (NDs) also increased the strength of AF orthodontic appliances [12]. Nevertheless, the NPs should be added after characterization and in a proper concentration to allow bonding with the resin matrix and avoid its agglomeration in clusters which could adversely affect the resin strength [11].

The surface roughness of denture base material is critical to avoid microbial colonization and staining, also adequate hardness makes the denture resist surface indentation. Alteration of surface properties with the addition of NPs to AF denture base resin was reported [11]. Gad et al. [11] found that incorporation of NS to AF denture resin increased the hardness though, the surface roughness was not changed.

The incorporation of NPs should enhance the mechanical performance of the resin, as well as its surface properties without altering the resin color. The denture base material should match the color of underlying gingiva and resist color change for acceptable esthetics. Studies that tested the effect of NS or ND on the color and surface properties of AF denture base resin are limited. Therefore, this study aimed to test the influence of NS or ND on the surface roughness, hardness, and color of two types of AF denture base resins. The study null hypothesis stated that incorporation of NPs would not alter the color or surface properties of AF denture base materials.

## 2. Materials and Methods

A total of 200 printed specimens (100 per resin, 50 for hardness testing, and 50 for Ra and color analysis, *n* = 10 for concentration) were fabricated based on the sample size calculation through the use of power analysis. The sample size was determined using the following parameters: power (80%), confidence interval (95%), and significance level (0.05). As a result, 10 was the estimated sample size for each group.

**Figure 1:**
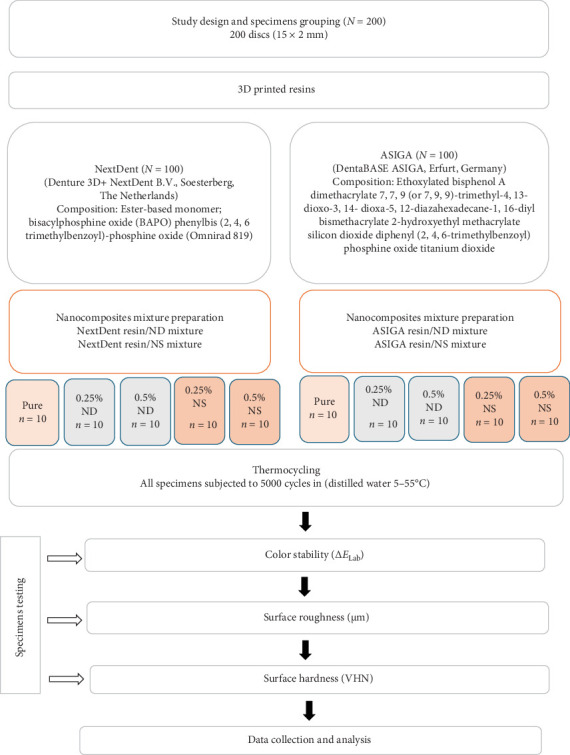
Study design.

Two AF denture base materials NextDent (Denture 3D + NextDent B.V., Soesterberg, The Netherlands) and ASIGA (DentaBASE ASIGA, Erfurt, Germany) were used for specimens printing. Each material was modified with NS or NDs in two concentrations (0.25% and 0.5%wt.), while one group remained unmodified as a control group (Figure 1).

### 2.1. Nanocomposite Preparation

Before adding NPs to fluid resins, surface treatment was completed for NS (AEROSIL R812; Evonik Degussa, Germany), using silane coupling agents while ND (Shanghai Richem International Co. Ltd, Shanghai, China) was treated with heat treatment as described in previous study [13] and the NPs were weighted using electronic balance. Each resin container was shacked using resin shaker (LC 3D Mixer, NextDent, Soesterberg, The Netherlands) for 1 h before NPs addition to allow the proper distribution of resin compositions as per the manufacturer recommendations. Then, the NPs were added to the shacked resins forming nanocomposite resins mixture that were furtherly stirred for 30 min for assurance of NPs distribution within the resin matrix.

### 2.2. Specimens Designing and Printing

Square shape specimen 10 mm × 10 mm × 2.5 mm was designed using opensource AutoCAD software (CAD software program, 123D design, Autodesk, version 2.2.14, California, USA) and was imported as standard tessellation language (STL) file to the printer of each material NextDent (NextDent 5100, NextDent B.V., Soesterberg, The Netherlands) and ASIGA (ASIGA MAX, ASIGA, Erfurt, Germany) with the printing order; 50 µm printing layer thickness and 90° printing angle [11]. The specimens were then cleaned from unpolymerized resin remnants using 99.9% isopropyl alcohol. The additional polymerization in post curing condition was completed according to manufacturer recommendation. After complete polymerization, all supporting structures were removed using bur followed by polishing with an automated polishing machine (Metaserv 250 grinder-polisher; Buehler GmbH, Lake Bluff, IL, USA) using 1200-grit sandpaper (MicroCut PSA; Buehler, IL, USA) for 5 min at 100 rpm in wet conditions, that was done by one investigator for polishing standardization. A digital caliper (SuperCaliper; Mitutoyo) was used to measure the specimens' dimensions. Specimens with different dimensions than the original designed ones or that included voids or defects seen by naked eye were discarded. The approved specimens were then kept for 2 days in distilled water at 37°C, followed by thermal cycling (THE-1100 Thermocycler, SD Mechatronik Thermocycler, Germany) for 5000 thermal cycles (5 and 55°C/30 s). The specimens were coded before testing by an investigator other than the one who performed the tests.

### 2.3. Color Change (∆*E*_Lab_) Measurement

A spectrophotometer (Color-Eye 7000A, X-Rite, Carlstadt, NJ, USA) in the visible spectrum (380–780 nm) was used for color measurements to obtain the values of color coordinates L^*⁣*^*∗*^^, a^*⁣*^*∗*^^, and b^*⁣*^*∗*^^. The color differences were calculated using the following equation: Δ*E*√((〖L^*∗*^〗_1_ − 〖L^*∗*^〗_2_)^2^+(〖a^*∗*^〗_1_ − 〖a^*∗*^〗_2_)^2^+〖(〖b^*∗*^〗_1_ − 〖b^*∗*^〗_2_)〗^2^), where the terms L_1_, a_1_, and b_1_ were the color values of the control group and L_2_, a_2_, and b_2_ the color values of the modified ones. The formula National Bureau of Standards (NBSs; NBS = ∆*E*_Lab_× 0.92) was used to compare color changes. A value over 1 NBS unit is detected by the human eye and up to 3.7 NBS unit is accepted esthetically and clinically, while values above 3.7 NBS unit are clinically undesirable [14, 15].

### 2.4. Hardness

The specimens Vickers hardness (VH) was tested using a hardness tester (HMV-2 Shimadzu Corp, Tokyo, Japan). The load applied was 50 g for 15 s dwell period and the average of the three readings for each specimen was recorded as the individual VH.

### 2.5. Surface Roughness

The surface roughness (Ra, µm) was measured by a noncontact profilometer (Contour GT-K 3D Optical Microscope, Bruker, Billerica, MA, USA). The Ra (arithmetical mean of the surface roughness) of three areas for each specimen was measured and the average was calculated.

### 2.6. Statistical Analysis

The data was normally distributed based on Shapiro Wilk test results, accordingly parametric tests were used for inferential analysis. The effect of categorical variables (with two categories) on the variation of mean was tested through the two-independent samples *T*-test. One-way ANOVA was used to compare the mean difference between the categorical variable with more than two categories followed by Tukey post hoc test. The interaction effects of type of material, NP, and concentration levels on the tested properties was evaluated by using three-way ANOVA. Statistically significance was established when *p*-value is less than 0.05.

## 3. Results

Three way-ANOVA results for all tested properties are summarized in Table 1. For color change (∆*E*_Lab_), the dual effect of material with NPs showed significant effect on the color however the rest of the combinations showed no statistical significance.

The interacting effect of the tested variables on hardness was examined by using three-way ANOVA, the effect of two variables (material with NP, material with concentration level, and NP with concentration level) significantly affected the hardness, though the joint effect of all three variables did not have any significant effect on the hardness.

Similarly, three-way ANOVA was used to evaluate the interacting effect of the variables on the roughness. The combined effect of the two variables (material with NP and NP with concentration level) had significant effect on the roughness. In addition, the combined effect of all three variables (material, NP, and concentration level) had also significant effect on the roughness.

The mean, SD, and significances of the color change between groups in relation to NP type and concentration are presented in Table 2. The color change was affected by the NP type. ND showed higher color change with NextDent in all groups (*p*  < 0.05) in comparison to NS. Whereas with ASIGA 0.25% NS showed higher color change than 0.25% ND (*p*=0.002) and the difference between ND and NS at 0.5% was not significant. The difference between the two tested concentrations per NP type on color change of both materials was not statistically significant. Comparing the color change between the two tested materials, NextDent and ASIGA, showed significant differences with all tested NPs. Color change of ASIGA with ND and NS was higher than that of NextDent. The color change values of all tested groups were below 3.7 NBS.

Table 3 summarized the mean, SD, and significances between groups in concern NP type and concentrations effect on the hardness of tested resins. For NextDent, when comparing both NPs at each concentration, NS showed higher hardness than ND at 0.25% and 0.5% (*p*=0.022 and *p*  < 0.001, respectively). Also, the hardness of NextDent was increased as ND (*p*=0.002) and NS (*p*  < 0.001) concentration increased. For ASIGA, hardness showed no significance differences between groups per NP addition as well as the concentrations between all groups. In concern material comparison, no significant differences were found in hardness between NextDent and ASIGA with ND, while 0.5% NS-NextDent showed significant increase when compared with ASIGA (*p*  < 0.001).

For NextDent the addition of ND showed no significant difference on Ra between tested groups (*p*=0.073). While NS addition showed significant differences between groups and 0.5% NS significantly showed the lowest Ra value (0.86 ± 0.2 µm). When comparing NP per %, only 0.5% NS showed significant decrease compared with 0.5%ND (*p*=0.001). For ASIGA, the addition of NPs showed no significate differences between all groups when compared concentrations and NP type (*p*  > 0.05). In term of materials comparison per NP type, significant differences between ASIGA and NextDent (*p*=0.01 for ND groups and *p*  < 0.001 for NS groups) with the variations between different concentrations. With ASIGA modified groups, only 0.25% had significantly the lowest Ra value (1.11 ± 0.2 µm) while 0.5% NS had the lowest Ra value (0.86 ± 0.2 µm) for NextDent (Table 4).

## 4. Discussion

The AF denture base resin in this study did not show significant color change after addition of NS and ND, while the surface roughness and hardness were altered by NP addition. Accordingly, the study null hypothesis is partially accepted.

The addition of fillers or nanofillers was recommended to improve the mechanical performance of denture base resins. However, the resin color could change with such addition and adversely affect the denture esthetics. The color change in this study was evaluated using CIE L^*⁣*^*∗*^^a^*⁣*^*∗*^^b^*⁣*^*∗*^^ which has been commonly used to evaluate color change of dental resins [16]. The color change value that resulted from CIE L^*⁣*^*∗*^^a^*⁣*^*∗*^^b^*⁣*^*∗*^^ formula was converted to NBS units to evaluate the extent of color change [14]. NBS units above 1 is perceived by naked eye while NBS unites above 3.7 is considered clinically unacceptable [15]. The color change caused by ND or NS with both concentrations was below the clinical acceptable value for all tested groups.

The present results showed that the material and NP type affected the color of the resin, while NP concentration had no significant effect on color change. The color change with ND and NS addition was more significant in ASIGA than NextDent. The difference in materials composition and the printing technology might be the cause of this variation. Previous studies noted difference in translucency and color stability between AF denture base resins after exposure to artificial aging, beverages, or denture cleansing [17, 18].

In addition, the color change caused by ND was higher than NS in NextDent. The effect of nanofillers on color change of AF denture base resin is not widely investigated; however, the effect of nanofiller on heat polymerized PMMA was tested before. The cause of color change by NP particles addition is due to the difference between the resin and NP refractive indices, leading to increased opacity of the nanocomposite [19]. The low difference between the refractive indices of NS (1.43) and resin matrix (1.48) could be the reason of the low perceived color change [13, 19].

The addition of ND with different concentrations showed no significant color change for both resins. In addition, all values were lower than the clinically acceptable value of 3.7 NBS unit. This finding may be related to the normal distribution of ND particles within the resin matrix even with the difference in the refractive index between ND (2.11) and resin (1.48) [13, 20]. Also, the low ND concentration tested in this study play a role and could be considered as a factor of the present results in comparison to the high ND concentrations (0.5%–5%) tested in a previous study [21]. The low concentrations permit proper ND distribution without clusters formation which resulted in color passing through the resin with high amount of color reflection rather than refraction. The finding of this study is in disagreement with a previous study that reported color change (5.24 ± 2.13) above the clinically perceptible value (Δ*E* > 3.3) with the addition of ND to denture base resin [21]. This variation in results may be related to the difference in materials used (PMMA vs. 3D printed) and variation in ND concentrations (5% and 2.5% vs. 0.25% and 0.5%) and storage solutions (distilled water vs. thermal cycling). Due to the lack of studies investigated the effect of the addition of ND to 3D printed resins, the comparison with previous studies was difficult. Therefore, the present findings should be explained with caution till further investigations confirm the findings of present study. However, recent studies reported significant change in the color of 3D printed denture base resin after the addition of titanium dioxide nanotubes (1.0% and 1.5%) or NPs (1 and 2 wt%). The variation between their findings and the present study results from testing different NPs and at higher concentrations [22, 23]. Similarly, Khattar et al. [24] found that addition of zirconium dioxide NPs at high concentration significantly decreased the translucency of 3D printed denture base resin.

The hardness results showed variation between both tested materials and between NP. ND and NS increased the hardness of NextDent showing higher values with higher concentration. Similarly Gad et al. [11] reported higher hardness of 3D printed resin modified with NS. Improved hardness with NS could have resulted from the proper distribution of the nanofiller within the resin matrix and bonding between the silanized NS and resin matrix [11]. The influence of ND on surface hardness of 3D printed resin was not tested previously to compare with the present results. However, previous studies found increased hardness of PMMA with ND addition support the present results [25, 26]. The increased hardness reported in this study might resulted from the low concentration of the added ND that allowed homogenous distribution within the resin matrix. Agglomeration of NPs at high concentration could deteriorate the mechanical properties of the resin rather than improving it [25, 27]. Moreover, ND has reactive oxide groups that enhance bonding with resin which might be the reason for increased hardness [28].

ND showed no effect on Ra of both resins which may be attributed to the small size of NP and good dispersions within resin matrix and specimens' surface. This in agreement with previous study showed that the incorporation of ND did not cause a significant difference in the Ra values of acrylic resin [21]. While NS showed a reduced Ra at 0.5% with NextDent, no change in Ra of ASIGA resin in both concentrations. Gad et al. [11], reported no change in surface roughness with NS addition to 3D printed resins and the change in values is due the printing layers and stepwise effects on the specimens' surface regardless the NP additions. Previous studies tested various NPs reported different results. Mhaibes et al. [22] found that addition of titanium dioxide nanotubes significantly decreased the surface roughness of 3D printable denture base resin. Also, Mohamed et al. [29] reported reduction in surface roughness of 3D printable denture base resin after addition of cerium oxide NPs due to the proper distribution of the NP filling the pores between the chains. While silver-loaded mesoporous silica NPs at concentration higher than 1wt.% increased the surface roughness of 3D printed denture base resin [30].

The added NPs showed various effect on the tested materials. The present results showed variation between the tested materials after addition of NPs, emphasizing the effect of material type on the tested properties. While the Ra and hardness of NextDent were altered by the addition of NPs, ASIGA showed no significant change of the aforementioned properties. Similarly, color change was different between NextDent and ASIGA with the addition of NPs. This is verified by the results of three-way ANOVA that showed significant effect of the combination of NP addition and material type on color change, Ra, and hardness of the tested materials. When comparing all NPs per resin and concentration, in ASIGA, no difference between NPs was found, this may be attributed to the composition of 3D printed resins and filler contents (Figure 1). NS and ND increased the hardness of NextDent at high concentration without negatively altering the color or Ra of the material. However, the NP concentration did not affect the tested properties of ASIGA in this study.

The present results revealed that the effect of NP on the tested properties is concentration dependent and material dependent. Accordingly, it is important to select the material along with the NP type and NP concentrations for better clinical performance of the 3D printed nanocomposite denture base. Hardness and surface roughness are important properties that affect the denture resistance to microbial adhesion and discoloration. Moreover, the color of denture base is required to match the adjacent mucosa for better esthetics. Therefore, it is important that addition of NP would deteriorate the surface properties of the denture base material and would not alter the color of the denture base material.

The specimens in this study were subjected to thermal cycling before testing to evaluate the effect of NP after artificial aging. However, among the study limitations is absence of other intraoral factors such exposure to oral flora, food and drinks, and various pH values due to the in vitro setting of this study, therefore, future in vivo studies are recommended. Future studies testing the color changes of AF nanocomposite after exposure to daily food and drinks and cleaning procedures need to be tested. In addition, the biocompatibility of AF with NPs needs to be evaluated and determining the proper concertation of each NP that does not cause adverse reaction.

## 5. Conclusions

Based on the findings of the present study the following is concluded:

• The color change of the tested AF denture base resins after addition of ND and NS was within the clinical acceptable range with both concentrations.
• The hardness of NextDent was increased with ND and NS, while with ASIGA the effect was not significant.
• Surface roughness was not altered by NP addition except 0.5% NS with NextDent. The tested properties showed variation between NextDent and ASIGA pointing out the influence of material type.

## Figures and Tables

**Table 1:** Three-way ANOVA results for all tested properties in term of nanoparticles, concentrations, and material combinations.

Tested properties	Source	Type III sum of squares	Df	Mean square	*F*-value	*p*-Value
∆*E*_Lab_	Intercept	450.853	1	450.853	632.433	<0.001^*∗*^
	Material × NP	17.456	1	17.456	24.486	<0.001^*∗*^
	Material × concentration	4.807	1	4.807	6.742	0.011^*∗*^
	NP × concentration	2.899	1	2.899	4.066	0.047^*∗*^
	Material × NP × concentration	0.186	1	0.186	0.261	0.611
	Error	51.328	72	0.713	—	—
	Total	574.537	80	—	—	—

Hardness (VHN)	Intercept	24,282.087	1	24,282.087	1544.554	<0.001^*∗*^
	Material × NP	165.543	1	165.543	10.530	0.002^*∗*^
	Material × concentration	258.912	1	258.912	16.469	<0.001^*∗*^
	NP × concentration	0.000	1	0.000	0.000	0.997
	Material × NP × concentration	29.330	1	29.330	1.866	0.176
	Error	1131.919	72	15.721	—	—
	Total	26,449.176	80	**—**	**—**	**—**

Surface roughness (Ra, µm)	Intercept	110.159	1	110.159	1538.961	<0.001^*∗*^
	Material × NP	0.023	1	0.023	0.323	0.572
	Material × concentration	0.323	1	0.323	4.510	0.037^*∗*^
	NP × concentration	0.902	1	0.902	12.599	0.001^*∗*^
	Material × NP × concentration	0.514	1	0.514	7.175	0.009^*∗*^
	Error	5.154	72	0.072	—	—
	Total	117.636	80	—	—	—

*⁣* ^*∗*^Statistical significance at 0.05 level of significance.

**Table 2:** Mean and SD of color change (∆*E*_Lab_) after addition of NP with different concentrations.

Groups NP (%)	Materials										ASIGA vs. NextDent
	NextDent (mean ± SD)					ASIGA (mean ± SD)					
	0.25%	NBS	0.5%	NBS	*p*	0.25%	NBS	0.5%	NBS	*p*	*p*
ND	1.83 (0.7)^A,B^	1.68	2.64 (1.0)	2.43	0.052	2.79 (0.6)^A^	2.57	2.81 (0.5)^B^	2.59	0.925	0.012^*∗*^
NS	0.89 (0.48)^A,B^	0.82	1.13 (0.59)^C,D^	1.03	0.331	3.91 (0.8)^A,C^	2.68	2.98 (0.5)^B,D^	2.74	0.11	<0.001^*∗*^
*p*	0.003^*∗*^	—	0.001^*∗*^	—	—	0.002^*∗*^	—	0.750	—	—	—

*Note:* Same superscript capital alphabets in each row showed statistical significance between materials. For NBS, differences above 3.7 NBS unit are rated a “mismatch” and considered as clinically unacceptable.

*⁣* ^*∗*^ indicates statistically significant difference.

**Table 3:** Mean and SD of hardness after addition of NP with different concentrations.

Groups NP (%)	Materials								ASIGA vs. NextDent
	NextDent (mean ± SD)				ASIGA (mean ± SD)				
	Pure	0.25%	0.5%	*p*	Pure	0.25%	0.5%	*p*	*p*
ND	14.58 (3.7)^a^	13.8 (2.3)^a^	18.7 (2.4)	0.002^*∗*^	14.1 (4.3)	15.4 (2.4)	15.5 (2.1)	0.516	0.059
NS	14.58 (3.7)^A^	18.59 (5.6)^B^	25.9 (4.6)	<0.001^*∗*^	14.1 (4.3)^A^	16.89 (4.6)^B^	14.6 (5.5)^A^	0.394	<0.001^*∗*^
*p*	—	0.022^*∗*^	<0.001^*∗*^	—	—	0.383	0.612	—	—

*Note:* Same superscript small alphabets in each row showed insignificant differences between the means per material. Same superscript capital alphabets in each row (between two materials) showed the insignificant difference between the means.

*⁣* ^*∗*^ indicates statistically significant difference.

**Table 4:** Mean and SD of surface roughness after addition of NP with different concentrations.

Groups NP (%)	Materials								ASIGA vs. NextDent	
	NextDent (mean ± SD)				ASIGA (mean ± SD)					
	Pure	0.25%	0.5%	*p*	Pure	0.25%	0.5%	*p*		*p*
ND	1.5 (0.24)^1^	1.21 (0.2)	1.37 (0.4)	0.073	1.57 (0.27)	1.11 (0.2)^1^	1.2 (0.3)	0.325		0.01^*∗*^
NS	1.5 (0.24)^a,1,2^	1.45 (0.3)^a,3,4^	0.86 (0.2)	<0.001^*∗*^	1.57 (0.27)	1.1 (0.2)^1,3^	1.1 (0.3)^2,4^	0.21		<0.001^*∗*^
*p*	—	0.073	0.001^*∗*^	—	—	0.876	0.381	—	—	

*Note:* Same superscript small alphabets in each row showed insignificant differences between the means. Same superscript numbers in each row (between two materials) showed the significant difference between the means.

*⁣* ^*∗*^ indicates statistically significant difference.

## Data Availability

All the study data are presented in the paper.
